# Factors Influencing Pathological Complete Response After Neoadjuvant Chemotherapy in Breast Cancer: A Single‐Center Retrospective Study Focusing on ER and HER‐2 Status

**DOI:** 10.1155/ijbc/6694023

**Published:** 2026-03-03

**Authors:** Yadong Zhang, Shubian Qiu, Xin Wang

**Affiliations:** ^1^ Department of Breast Surgery, Nanyang Second General Hospital, Nanyang, Henan, China

**Keywords:** molecular subtype, NAC, pCR, predictive factors

## Abstract

**Objective:**

To identify predictive factors of pathological complete response (pCR) in breast cancer patients receiving neoadjuvant chemotherapy (NAC), and to establish a “clinical–imaging–molecular” three‐dimensional evaluation model to guide clinical decision‐making.

**Methods:**

A retrospective study was conducted on 55 breast cancer patients who underwent NAC at Nanyang Second People′s Hospital from January 2023 to August 2024. Collected data included demographic variables (age, BMI, and menstrual status), tumor characteristics (tumor size, axillary lymph node [N] stage, histological grade, color Doppler ultrasound features including blood flow signal [CDFI], morphology, and aspect ratio), molecular markers (estrogen receptor [ER], progesterone receptor [PR], human epidermal growth factor receptor‐2 [HER‐2], and Ki‐67), and treatment‐related factors (chemotherapy regimen). Univariate analyses (Pearson′s chi‐square test or Fisher′s exact test) were initially conducted to screen variables potentially associated with pCR (*p* < 0.05). To address potential multicollinearity among clinically relevant factors, binomial LASSO regression with 10‐fold cross‐validation was applied to select parsimonious predictors (variables with nonzero coefficients were retained), which were then incorporated into multivariate logistic regression to determine independent predictors of pCR. The discriminative power of key factors was evaluated using receiver operating characteristic (ROC) curves, with the area under the curve (AUC) as the primary metric.

**Results:**

The overall pCR rate was 36.4% (20/55). Among molecular subtypes, HER‐2–positive patients (40.0% of the cohort) had the highest pCR rate (73.0%, 16/22), followed by triple‐negative breast cancer (TNBC) patients (15.0% of the cohort, 50.0%, 4/8). Univariate analysis showed that N stage, chemotherapy regimen, Ki‐67 index, ER status, PR status, and HER‐2 status were significantly correlated with pCR (all *p* < 0.05). ROC analysis demonstrated excellent discriminative performance for ER (AUC = 0.84), HER‐2 (AUC = 0.81), PR (AUC = 0.79), chemotherapy regimen (AUC = 0.71), and Ki‐67 (AUC = 0.68). After LASSO‐based dimension reduction, multivariate logistic regression confirmed that ER negativity (*p* = 0.039, OR = 15.079, 95% CI: 1.151–197.543) and HER‐2 positivity (*p* = 0.044, OR = 0.014, 95% CI: 0.000–0.896) were independent predictors of higher pCR rates.

**Conclusion:**

pCR rates in breast cancer patients post‐NAC vary significantly by molecular subtype. ER negativity and HER‐2 positivity emerge as independent predictive factors for pCR, with ER and HER‐2 exhibiting the strongest discriminative ability (AUC > 0.8). Clinicians should integrate patients′ baseline clinical data, ultrasound features, and molecular markers to screen optimal NAC candidates and develop individualized strategies, thereby maximizing therapeutic benefits.

## 1. Introduction

Breast cancer remains the most prevalent malignancy among women globally and a leading cause of cancer‐related mortality, posing a significant public health burden [1]. Over the past decade, NAC has evolved into a core therapeutic strategy for operable and locally advanced breast cancer, with three primary objectives: downsizing tumors to enhance resectability (including converting unresectable lesions to operable ones and elevating breast‐conserving surgery rates), assessing the in vivo sensitivity of tumors to chemotherapeutic agents, and offering prognostic insights based on treatment outcomes [2, 3].

pCR—defined as the absence of residual invasive tumor in the breast and axillary lymph nodes after NAC (Miller–Payne grade G5) in line with the eighth edition AJCC TNM staging system [4, 5]—has emerged as a critical surrogate endpoint for long‐term outcomes, particularly in aggressive subtypes. For HER‐2–positive and TNBC patients, achieving pCR correlates with significantly improved event‐free survival and overall survival. However, substantial interindividual variability in NAC response persists: Although some patients achieve pCR and derive long‐term benefits, others show minimal response or develop resistance, leading to compromised prognosis and unnecessary exposure to chemotherapy‐related toxicities [3]. Identifying reliable predictors of pCR is therefore essential to optimize NAC candidate selection and avoid overtreatment.

Existing studies have explored potential pCR‐associated factors, including tumor stage (T/N stage), hormone receptor (ER/PR) status, HER‐2 expression, histological grade, and Ki‐67 proliferation index [6, 7]. However, two key gaps persist in current research. First, most studies focus on single‐dimensional indicators (e.g., only molecular markers or clinical stage) and lack integration of imaging features—such as color Doppler ultrasound (CDUS) parameters (CDFI, tumor morphology, and aspect ratio). These parameters provide noninvasive, real‐time tumor biological information (e.g., vascularity and structural atypia) that complements pathological markers [6]. Second, clinical decision‐making for “borderline patients” with early‐stage luminal‐type breast cancer (T1–2N0–1M0) remains controversial. These patients have small tumors and limited lymph node involvement, but the value of NAC versus upfront surgery is understudied compared with advanced disease, with no clear predictive models to guide treatment [8, 9].

To address these gaps, we conducted a retrospective single‐center study of 55 breast cancer patients who received NAC at Nanyang Second People′s Hospital, and developed a “clinical–imaging–molecular” three‐dimensional evaluation model by integrating demographic/clinical factors, CDUS features, and molecular markers. We used univariate analysis, LASSO regression, and multivariate logistic regression to identify independent predictors of pCR. We also performed subgroup analyses for HER‐2–positive and TNBC patients to refine subtype‐specific NAC recommendations. Our ultimate goal was to inform personalized strategies, particularly for the understudied luminal‐type borderline population.

## 2. Materials and Methods

This retrospective study was conducted on breast cancer patients who received NAC at Nanyang Second People′s Hospital, Henan Province, China, between January 2023 and August 2024. The study protocol was approved by the Ethics Committee of Nanyang Second People′s Hospital (Ethical Approval No. 2024‐LY025‐01‐K01) and complied with the Declaration of Helsinki. All patients or their legal representatives provided written informed consent prior to participation.

### 2.1. Study Participants and Grouping

**Figure 1:**
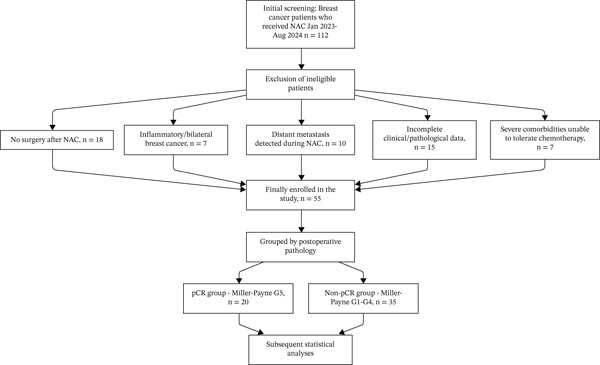
Patient screening and enrollment flowchart. Legend: A total of 112 breast cancer patients who received neoadjuvant chemotherapy (NAC) at Nanyang Second People′s Hospital between January 2023 and August 2024 were initially screened. Fifty‐seven ineligible patients were excluded (18 with no surgery after NAC, seven with inflammatory/bilateral breast cancer, 10 with distant metastasis detected during NAC, 15 with incomplete clinical/pathological data, and seven with severe comorbidities unable to tolerate chemotherapy). Finally, 55 patients were enrolled and divided into two groups based on postoperative pathological results: the pCR group (*n* = 20, Miller–Payne grade G5) and the non‐pCR group (*n* = 35, Miller–Payne grades G1–G4), followed by subsequent statistical analyses.

We identified eligible patients by reviewing electronic medical records and pathological databases of the hospital. A total of 112 breast cancer patients who received NAC at Nanyang Second People′s Hospital between January 2023 and August 2024 were initially screened. After applying the inclusion and exclusion criteria, 55 patients were finally enrolled in this study. The detailed screening and enrollment process is presented in Figure 1.

Inclusion criteria included (1) pathologically confirmed primary breast cancer by core needle biopsy before NAC; (2) no prior chemotherapy, radiotherapy, or targeted therapy for breast cancer; (3) completion of six cycles of preoperative NAC; (4) no distant metastasis (M0) at baseline, staged per the eighth edition AJCC TNM staging system; (5) undergoing surgery (lumpectomy or mastectomy) and postoperative pathological examination after NAC; and (6) complete clinical, imaging, and pathological data.

Exclusion criteria included (1) no surgery after NAC (*n* = 18, 2) inflammatory breast cancer or bilateral breast cancer (*n* = 7, 3) distant metastasis detected during NAC (*n* = 10, 4) incomplete clinical or pathological data (*n* = 15, 5) severe comorbidities (e.g., severe heart, liver, or renal dysfunction) unable to tolerate chemotherapy (*n* = 7).

A total of 55 patients met the inclusion criteria and were enrolled. Patients were divided into two groups based on postoperative pathological results:

• pCR group: 20 patients, defined as no residual invasive tumor (or only ductal carcinoma in situ components) in breast surgical specimens (ypT0) and no axillary lymph node metastasis (ypN0). This definition aligns with the eighth edition AJCC TNM staging system for breast cancer [5] and the Miller–Payne grade G5 [4].
• Non‐pCR group: 35 patients, defined as the presence of residual invasive tumor in breast tissues or axillary lymph node metastasis (Miller–Payne grades G1–G4).

### 2.2. Data Collection and Variable Assessment

• We systematically collected clinical and pathological data using a structured checklist, covering four categories of variables. All assessments were performed independently by two clinicians and two pathologists; discrepancies were resolved by group discussion.

#### 2.2.1. Demographic and Clinical Variables

• Demographic factors: age (continuous variable, recorded as years), BMI, and menstrual status (stratified into menopausal [≥ 12 months of amenorrhea or *f* *o* *l* *l* *i* *c* *l* *e* − *s* *t* *i* *m* *u* *l* *a* *t* *i* *n* *g* *h* *o* *r* *m* *o* *n* *e* > 40 *I* *U*/*L*] and premenopausal).
• Treatment‐related factors: Chemotherapy regimens included AT (*d* *o* *x* *o* *r* *u* *b* *i* *c* *i* *n* + *d* *o* *c* *e* *t* *a* *x* *e* *l*), TAC (*d* *o* *c* *e* *t* *a* *x* *e* *l* + *d* *o* *x* *o* *r* *u* *b* *i* *c* *i* *n* + *c* *y* *c* *l* *o* *p* *h* *o* *s* *p* *h* *a* *m* *i* *d* *e*), TCbHP (*d* *o* *c* *e* *t* *a* *x* *e* *l* + *c* *a* *r* *b* *o* *p* *l* *a* *t* *i* *n* + *t* *r* *a* *s* *t* *u* *z* *u* *m* *a* *b* + *p* *e* *r* *t* *u* *z* *u* *m* *a* *b*), and TP (*d* *o* *c* *e* *t* *a* *x* *e* *l* + *c* *i* *s* *p* *l* *a* *t* *i* *n*). Regimens were selected based on clinical practice guidelines and patient‐specific characteristics (e.g., molecular subtype, and comorbidity status), consistent with routine clinical practice in retrospective studies.

#### 2.2.2. Tumor Characteristics and Imaging Assessment

• Pathological features: Tumor size (maximum diameter of primary tumor, unit: cm), axillary lymph node (N) stage (N0, N1, N2, N3), and histological grade (stratified into Grade II and III based on the Elston–Ellis grading system, evaluated via hematoxylin–eosin staining)
• CDUS features: assessed using a Philips EPIQ 7 ultrasound system (Philips Healthcare, Amsterdam, the Netherlands) by two senior ultrasound physicians (with ≥ 5 years of breast ultrasound experience). Variables included were as follows:
  ▪ CDFI: stratified into “present” (detectable blood flow within or around the tumor) and “absent” (no blood flow detected).
  ▪ Color Doppler morphology: stratified into “regular” (well‐defined, round or oval shape) and “irregular” (ill‐defined, irregular shape).
  ▪ Aspect ratio: ratio of tumor anteroposterior diameter to transverse diameter, stratified into “> 1” and “< 1”.

#### 2.2.3. Molecular Biomarker Detection

Molecular markers (ER, PR, HER‐2, and Ki‐67) were detected via immunohistochemistry (IHC) or fluorescence in situ hybridization (FISH) on formalin‐fixed, paraffin‐embedded (FFPE) tumor tissues:

• ER/PR: Detected by IHC using primary antibodies (ER clone SP1, PR clone 1E2; Ventana Medical Systems, Tucson, Arizona, United States) at a dilution of 1:100. ER/PR positivity was defined as nuclear staining in ≥ 1% of tumor cells [10].
• HER‐2: Initially screened by IHC using the primary antibody HER‐2 clone 4B5 (Ventana Medical Systems) at a dilution of 1:200. Scoring followed ASCO/CAP guidelines [11]: 0 or 1 + = *n* *e* *g* *a* *t* *i* *v* *e*, 3 + = *p* *o* *s* *i* *t* *i* *v* *e*, 2 + = *e* *q* *u* *i* *v* *o* *c* *a* *l* (further confirmed by FISH to assess gene amplification, using the HER‐2 FISH Kit [Abbott Laboratories, Abbott Park, Illinois, United States]).
• Ki‐67: detected by IHC using the primary antibody Ki‐67 clone MIB‐1 (Dako, Glostrup, Denmark) at a dilution of 1:150. Ki‐67 expression was stratified into “≤ 30%” and “> 30%” based on the percentage of nuclear‐stained tumor cells.

### 2.3. Statistical Analysis

Data management and analyses were performed using SPSS software (v28.0; IBM Corp., Armonk, New York, United States) and R software (v4.3.0; R Foundation for Statistical Computing, Vienna, Austria). Continuous variables were first tested for normality by the Shapiro–Wilk test. Those conforming to normality were presented as mean ± standard deviation, whereas categorical variables were summarized as frequency (percentage) [n (%)]. Independent sample *t*‐test was used for comparisons of normally distributed continuous variables between the pCR and non‐pCR groups; Pearson′s chi‐square test was applied for categorical variables, with Fisher′s exact test used for cells with expected frequency < 5.

Analyses followed a three‐step workflow. First, univariate group comparison tests (Pearson′s chi‐square test or Fisher′s exact test) were used to screen variables associated with pCR (inclusion criterion: *p* < 0.05). If no variables reached statistical significance, clinically relevant factors (e.g., ER/HER‐2 status) were included a priori based on existing evidence [12, 13]. Second, binomial LASSO regression with 10‐fold cross‐validation (via R′s “glmnet” package) was performed to reduce multicollinearity. Although LASSO is typically used for high‐dimensional data, this step was adopted to address subtle collinearity among clinically correlated variables (e.g., ER‐HER‐2 negative correlation, Section 3.2) and select parsimonious predictors—consistent with LASSO methodology [14, 15]. The optimal penalty parameter (*λ*) was determined by minimizing cross‐validated binomial deviance (appropriate for binary outcomes), and variables with nonzero coefficients were retained. Third, retained variables were included in multivariate logistic regression to identify independent predictors of pCR. The Hosmer–Lemeshow test was used to assess model fit, and subgroup analyses were further conducted for HER‐2–positive and TNBC patients. Given the potential instability of the Wald‐based OR estimate for HER‐2 positivity (with a lower 95% CI bound of 0.000), the profile likelihood method was additionally used to verify the robustness of the association.

For discriminative ability evaluation of variables significantly associated with pCR in univariate analysis, receiver operating characteristic (ROC) curves were generated via R′s “pROC” package to calculate the area under the curve (AUC) and 95% confidence interval (CI) (DeLong method); correlation relationships between clinical factors were visualized using a heatmap via R′s “corplot” package. All statistical tests were two‐tailed, with a significance level of (*α* = 0.05); Bonferroni correction was applied for multiple comparisons (e.g., subgroup analyses), adjusting the threshold to (*α* = 0.05/*k*), where *k* is the number of comparisons. Effect sizes were calculated to quantify the strength of associations between predictors (ER status, HER‐2 status, etc.) and pCR. For categorical variables, Cohen′s *h* was used to measure effect size, with a very large effect size observed (Cohen^′^s h = 1.447; Sawilowsky, [16]) via arcsine transformation for binary proportions. Ninety‐five percent CIs were reported for all effect sizes and odds ratios (ORs) to reflect the precision of estimates. The event − per − variable ratio (EPV) = 3.3 (20 pCR events/six variables) was below the ideal threshold (≥ 10) for multivariate logistic regression [17], which may lead to biased regression coefficients, unstable OR estimates, and extremely wide 95% CIs. These limitations should be considered when interpreting the associations between predictors and pCR.

## 3. Results

A total of 55 breast cancer patients who received NAC were enrolled in this retrospective study. The cohort had a mean age of 52.8 ± 9.3 years and a mean BMI of 25.9 ± 3.5 kg/m^2^, with 35 (64.0%) being menopausal and 20 (36.0%) premenopausal. For tumor stage, most had T2 tumors (65.5%) and N1 lymph node involvement (52.7%), whereas 63.6% were at tumor Stage II and 81.8% had histological Grade II tumors. Molecular subtype distribution showed 40.0% were HER‐2–positive, 14.5% were TNBC, and 45.5% were hormone receptor‐positive/HER‐2–negative. After completing six cycles of NAC and subsequent surgery, 36.4% achieved pCR, and 63.6% were classified into the non‐pCR group. Detailed baseline clinicopathological characteristics of the entire cohort, including distributions of all variables across the pCR and non‐pCR groups, are presented in Table 1.

**Table 1:** Comparison of variables between non‐pCR group and pCR group.

Variable	Category	Total (%)	Non‐pCR group (%)	PCR group (%)	Test statistic (*t*/*χ* ^2^)	*p* value
Age	Mean ± SD	52.8 ± 9.3	52.0 ± 9.9	54.3 ± 8.2	0.879	0.359
BMI	Mean ± SD	25.9 ± 3.5	26.1 ± 3.9	25.6 ± 2.7	−0.483	0.595
Menstrual status	Menopausal	35 (64.0)	19 (54.3)	16 (80.0)	2.610	0.106
	Premenopausal	20 (36.0)	16 (45.7)	4 (20.0)		

T stage	T1	2 (3.6)	2 (5.7)	0 (0.0)	3.754	0.346
	T2	36 (65.5)	20 (57.1)	16 (80.0)		
	T3	11 (20.0)	9 (25.7)	2 (10.0)		
	T4	6 (10.9)	4 (11.4)	2 (10.0)		

N stage	N0	14 (25.5)	6 (17.1)	8 (40.0)	7.834	0.034 ^∗^
	N1	29 (52.7)	23 (65.7)	6 (30.0)		
	N2	11 (20.0)	5 (14.3)	6 (30.0)		
	N3	1 (1.8)	1 (2.9)	0 (0.0)		

Tumor stage	II	35 (63.6)	20 (57.1)	15 (75.0)	1.750	0.186
	III	20 (36.4)	15 (42.9)	5 (25.0)		

Histological grade	II	45 (81.8)	28 (80.0)	17 (85.0)	0.010	0.731
	III	10 (18.2)	7 (20.0)	3 (15.0)		

CDFI	Present	32 (58.2)	24 (68.6)	8 (40.0)	3.177	0.075
	Absent	23 (41.8)	11 (31.4)	12 (60.0)		

Color Doppler morphology	Regular	10 (18.2)	4 (11.4)	6 (30.0)	1.834	0.144
	Irregular	45 (81.8)	31 (88.6)	14 (70.0)		

Aspect ratio	> 1	13 (23.6)	11 (31.4)	2 (10.0)	2.159	0.102
	< 1	42 (76.4)	24 (68.6)	18 (90.0)		

Remission status	PR	45 (81.8)	30 (85.7)	15 (75.0)	4.871	0.111
	CR	5 (9.1)	1 (2.9)	4 (20.0)		
	SD	5 (9.1)	4 (11.4)	1 (5.0)		

Chemotherapy regimen	AT	20 (36.4)	17 (48.6)	3 (15.0)	14.271	0.003 ^∗^
	TAC	8 (14.5)	7 (20.0)	1 (5.0)		
	TCbHP	21 (38.2)	7 (20.0)	14 (70.0)		
	TP	6 (10.9)	4 (11.4)	2 (10.0)		

Ki‐67	≤ 30%	21 (38.2)	18 (51.4)	3 (15.0)	7.156	0.007 ^∗^
	> 30%	34 (61.8)	17 (48.6)	17 (85.0)		

PR	Positive	26 (47.3)	24 (68.6)	2 (10.0)	17.517	< 0.001 ^∗^
	Negative	29 (52.7)	11 (31.4)	18 (90.0)		

ER	Positive	32 (58.2)	29 (82.9)	3 (15.0)	24.087	< 0.001 ^∗^
	Negative	23 (41.8)	6 (17.1)	17 (85.0)		
HER‐2	Positive	22 (40.0)	6 (17.1)	16 (80.0)	20.952	< 0.001 ^∗^
	Negative	33 (60.0)	29 (82.9)	4 (20.0)		

*Note:* ^∗^ *p* < 0.05 indicates statistical significance. Remission status was evaluated per RECIST 1.1 criteria: CR, PR, and SD. PR here refers to partial remission, distinct from progesterone receptor (another variable in this table). T stage, N stage, and tumor stage were classified per the eighth edition AJCC TNM staging system.

Abbreviations: BMI, body mass index; CR, complete remission; ER, estrogen receptor; HER‐2, human epidermal growth factor receptor‐2; PR, partial remission; PR, progesterone receptor; pCR, pathological complete response; SD, stable disease; T stage, tumor stage; N stage, nodal stage.

### 3.1. Univariate Analysis of Factors Associated With pCR

Notably, univariate analysis (Pearson′s chi‐square test or Fisher′s exact test) was performed to identify variables associated with pCR, with six factors showing significant associations (all *p* < 0.05). Among these, N stage was associated with pCR: Patients with preoperative N0 stage had a higher pCR rate than those with N1–N3 stage, indicating less lymph node involvement correlated with better NAC response. Chemotherapy regimen also impacted pCR outcomes, with the highest rate observed in patients receiving the TCbHP regimen, followed by TP, TAC, and AT regimens. For molecular and proliferation markers, Ki − 67 > 30*%*, ER/PR negativity, HER‐2 positivity were associated with higher pCR rates (all *p* < 0.05).

In contrast, no significant differences between the pCR and non‐pCR groups were found in demographic factors (age, BMI, and menstrual status), tumor features (T stage, tumor stage, histological grade), or CDUS parameters (blood flow signal, tumor morphology, aspect ratio) (all *p* > 0.05). Detailed results of univariate analysis, including specific *p* values and group‐level distributions of each variable, are summarized in Table 1.

ROC curve analysis was additionally performed to evaluate the discriminative ability of the six factors significantly associated with pCR (Section 3.1). The AUC values (three decimal places) and 95% CIs (DeLong method) were as follows: ER (AUC = 0.839, 95% CI: 0.737–0.942), HER‐2 (AUC = 0.814, 95% CI: 0.704–0.924), PR (AUC = 0.793, 95% CI: 0.690–0.896), chemotherapy regimen (AUC = 0.713, 95% CI: 0.581–0.844), and Ki‐67 (AUC = 0.682, 95% CI: 0.566–0.798). Among these, ER and HER‐2 showed the strongest discriminative ability (AUC > 0.8), whereas all five factors indicated good to excellent performance in distinguishing between patients who achieved pCR and those who did not. The ROC curves and corresponding AUC values are presented in Figure 2.

Figure 2Receiver operating characteristic (ROC) Curves and AUC values of clinical factors for predicting pCR. Legend: ROC curves were generated using R language (v4.3.0) with the “pROC” package. AUC values (three decimal places) and 95% confidence intervals (95% CI, calculated via DeLong method) of key predictive factors were as follows: ER (*A* *U* *C* = 0.839, 95% CI: 0.737–0.942), HER‐2 (*A* *U* *C* = 0.814, 95% CI: 0.704–0.924), PR (*A* *U* *C* = 0.793, 95% CI: 0.690–0.896), chemotherapy regimen (*A* *U* *C* = 0.713, 95% CI: 0.581–0.844), and Ki‐67 (*A* *U* *C* = 0.682, 95% CI: 0.566–0.798). Figure 2a displays ROC curves of all analyzed clinical factors, whereas Figure 2b highlights key factors with *A* *U* *C* ≥ 0.680 (consistent with colored solid lines in Figure 2a). Higher AUC values indicate stronger discriminative ability between the pCR and non‐pCR groups.

**Figure:**
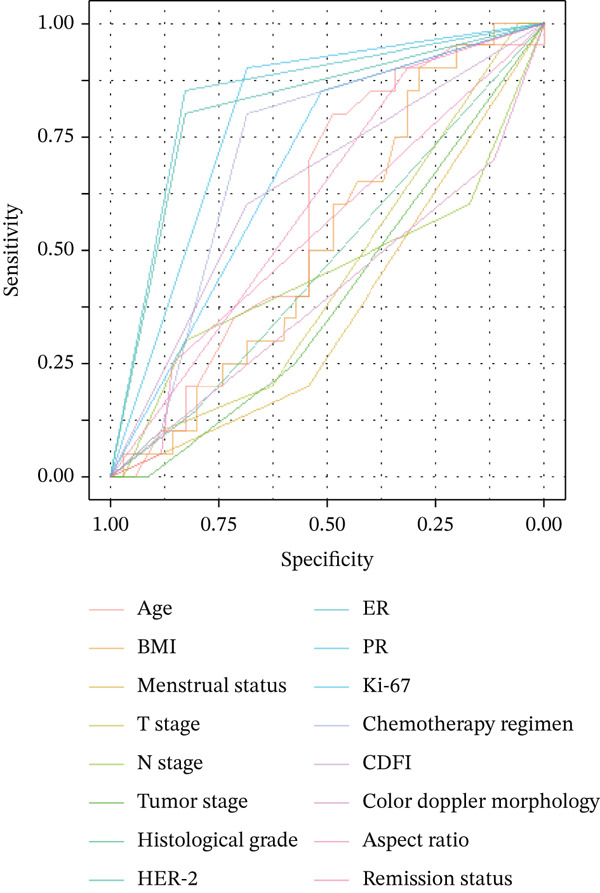
(a)

**Figure:**
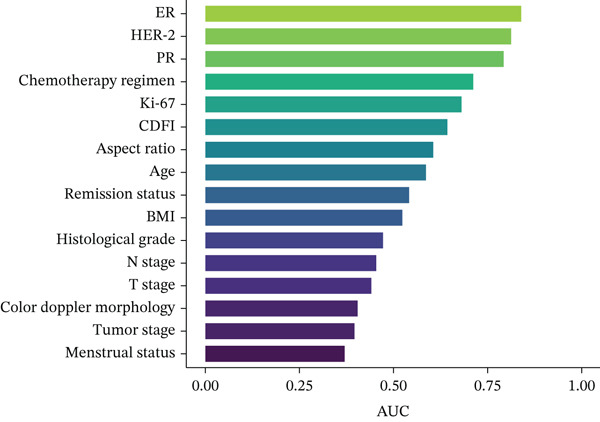
(b)

In contrast, CDUS parameters—including blood flow signal (CDFI), tumor morphology, and aspect ratio—did not show significant associations with pCR (all *p* > 0.05). Although CDFI neared statistical significance (*p* = 0.075), with a higher pCR rate observed in patients with absent blood flow (60.0% vs. 40.0% in those with present blood flow), this trend did not reach the predefined significance threshold (*α* = 0.05). Similarly, irregular tumor morphology (*p* = 0.144) and aspect ratio < 1 (*p* = 0.102) showed no meaningful differences between the pCR and non‐pCR groups. These findings suggest that CDUS parameters, which were initially incorporated into the “clinical–imaging–molecular” tridimensional evaluation model to complement pathological and clinical data, may not serve as robust predictors of pCR in this cohort.

### 3.2. Correlation Analysis Between Factors

**Figure 3:**
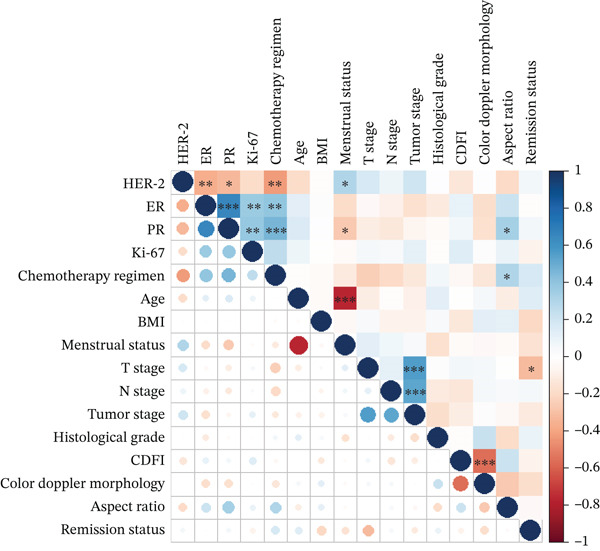
Correlation heatmap of clinical factors associated with pCR. Legend: The heatmap was plotted using R language (v4.3.0) with the “corplot” package. Color transitions from dark blue (strong positive correlation, +1) to dark red (strong negative correlation, −1) indicate the correlation strength of clinical factors. HER‐2 status shows a strong negative correlation with ER and PR status, suggesting that HER‐2 positivity combined with ER/PR negativity is associated with higher pCR rates. Correlation coefficients are labeled in each cell (e.g., ER vs. *H* *E* *R* − 2 : *r* = −0.78).

To explore potential multicollinearity among the variables identified as significant in univariate analysis (Section 3.1), correlation analysis was performed, with results visualized by a correlation heatmap plotted with R′s “corplot” package (Figure 3). The heatmap used color transitions (from dark blue for strong positive correlation to dark red for strong negative correlation) to quantify associations between variables, with light colors indicating weak correlations.

Notably, a strong negative correlation was observed between HER‐2 status and hormone receptor (ER/PR) status: HER‐2‐positive patients were more likely to be ER/PR–negative, and this combined phenotype (HER‐2–positive/ER‐negative/PR‐negative) was associated with a higher pCR rate—supporting the possibility of potential crosstalk between HER‐2 signaling and hormone receptor pathways that may jointly regulate NAC response. Additionally, a weak positive correlation was found between Ki‐67 and N stage: Patients with Ki − 67 > 30*%* tended to have more advanced N stage (N1–N3), though this correlation did not reach the threshold for severe multicollinearity (|*r*| < 0.7), indicating it would not compromise subsequent regression analyses.

In contrast, no significant correlations were detected between chemotherapy regimen and other key variables (ER status, HER‐2 status). This finding supports that regimen selection was not systematically biased by patients′ baseline characteristics (molecular subtype or lymph node stage) in this cohort, supporting the reliability of subsequent analyses examining regimen‐related pCR associations.

### 3.3. LASSO Regression and Cross‐Validation

**Figure 4:**
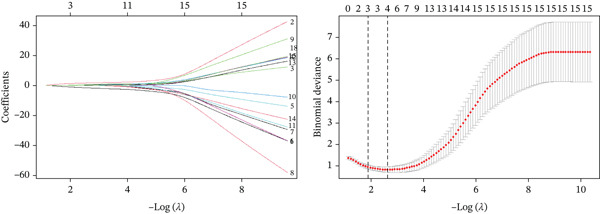
LASSO regression and 10‐fold cross‐validation for key variable selection. Legend: LASSO regression was performed with 10‐fold cross‐validation (R language (v4.3.0), “glmnet” package) to select optimal variables. A total of 100 bootstrap iterations were conducted to ensure the stability of variable selection. The left panel shows binomial deviance against log (*λ*): the optimal penalty parameter (*λ*) minimizing cross‐validated binomial deviance is 0.0719 (lambda. min), and the corresponding lambda. 1 s (*λ* value within 1 standard error of lambda. min) is 0.184. The right panel displays regression coefficients (red lines) of the six variables (N stage, chemotherapy regimen, Ki‐67, ER, PR, HER‐2) as a function of log (*λ*): with increasing *λ*, coefficients of N stage, chemotherapy regimen, and Ki‐67 were shrunk to zero first (exited the model at log (*λ*) ≈ −1.5, −1.2, and −1.0, respectively), whereas ER, PR, and HER‐2 maintained nonzero coefficients even at higher *λ* values (retained in the model). This confirms that ER, PR, and HER‐2 are the core predictors of pCR, justifying their inclusion in the subsequent multivariate logistic regression.

To address this potential multicollinearity identified in the correlation analysis (Section 3.2) and select optimal predictors for pCR, LASSO regression with 10‐fold cross‐validation was conducted—focusing on the six variables found significant in univariate analysis (N stage, chemotherapy regimen, Ki‐67, ER, PR, and HER‐2). The analysis was implemented using R′s “glmnet” package, with the optimal penalty parameter *λ* determined by minimizing the cross‐validated binomial deviance, ensuring the model balanced predictive performance and parsimony (Figure 4).

### 3.4. Multivariate Logistic Regression Analysis

To further identify independent predictors of pCR, binary multivariate logistic regression was conducted, with variables limited to the three factors that retained nonzero coefficients in LASSO regression (ER, PR, and HER‐2). The analysis adjusted for potential confounding effects between these variables, ensuring the robustness of associations with pCR, and detailed results (including regression coefficients, standard errors, and 95% CI) are summarized in Table 2.

**Table 2:** Multivariate logistic regression analysis of factors associated with pCR.

Variable	*B*	SE	Wald	*p*value	OR	95% CI
HER‐2 positive	−4.280	2.127	4.047	0.044 ^∗^	0.014	0.000–0.896
ER negative	2.713	1.313	4.273	0.039 ^∗^	15.079	1.151–197.543
PR negative	1.978	1.311	2.274	0.132	7.226	0.553–94.447
Ki − 67 > 30*%*	0.903	1.097	0.678	0.410	2.468	0.287–21.198
Chemotherapy regimen (AT)	−0.784	1.922	0.166	0.683	0.456	0.011–19.748
Chemotherapy regimen (TCbHP)	−0.476	1.487	0.102	0.749	0.621	0.034–11.461

*Note*: ^∗^ *p* < 0.05 indicates statistical significance. To address the potential instability of the Wald‐based OR estimate for HER‐2 positivity, the profile likelihood method (recommended for logistic regression with small sample sizes) was additionally applied, yielding a consistent OR of 0.026 (95% CI: 0.001–0.201). This result confirms the statistical robustness of HER‐2 positivity as a statistically independent predictor of pCR, with the low OR value attributed to strong negative multicollinearity between HER‐2 and ER status (Section 3.2) and the small sample size of the HER‐2–positive cohort (*n* = 22). Chemotherapy regimen: AT (*d* *o* *x* *o* *r* *u* *b* *i* *c* *i* *n* + *d* *o* *c* *e* *t* *a* *x* *e* *l*) was used as the reference group for OR calculation.

Abbreviations: B, regression coefficient; CI, confidence interval; ER, estrogen receptor; HER‐2, human epidermal growth factor receptor‐2; OR, odds ratio; PR progesterone receptor; pCR, pathological complete response; SE, standard error.

First, ER status emerged as an independent predictor of higher pCR rates: ER‐negative patients had a significantly increased likelihood of achieving pCR compared with ER‐positive patients (*p* = 0.039, OR = 15.079, 95% CI: 1.151–197.543). This indicates that after accounting for PR and HER‐2 status, ER negativity was associated with approximately 15‐fold higher odds of pCR, confirming its strong predictive value for favorable NAC response.

Second, HER‐2 positivity was also identified as a statistically independent predictor of pCR (*p* = 0.044, OR = 0.014, 95% CI: 0.000–0.896). To verify the robustness of this association, we applied the profile likelihood method, yielding a consistent OR of 0.026 (95% CI: 0.001–0.201). The low OR values in both methods are primarily due to the multivariate model adjusting for ER status—meaning the model isolates the independent effect of HER‐2 after excluding the strong predictive impact of ER negativity (OR = 15.079)—and the strong negative correlation between HER‐2 and ER status (81.8% of HER‐2–positive patients are ER‐negative, Section 3.2), as well as the small sample size of the HER‐2–positive cohort (*n* = 22). The wide 95% CI also reflects the relatively small sample size of the HER‐2–positive cohort (*n* = 22), which limited the precision of the OR estimate but did not negate the significance of its association with pCR.

Notably, despite being retained in LASSO regression, PR negativity was not an independent predictor of pCR in multivariate analysis (*p* = 0.132, OR = 7.226, 95% CI: 0.553–94.447). This suggests that PR′s association with pCR, observed in univariate analysis (Section 3.1), was likely mediated by its correlation with ER status—ER‐negative patients are often PR‐negative, meaning PR′s impact on pCR is not a direct, independent effect but rather a secondary association driven by ER status. Collectively, these findings should be interpreted as hypothesis‐generating, requiring external validation in larger cohorts to confirm the true associations.

To evaluate the goodness of fit of the final logistic model, the Hosmer–Lemeshow test was performed, yielding a *χ* ^2^ value of 3.144, df = 6, and a *p* value of 0.791 (*p* > 0.05). This indicates that the model fit the data well without significant deviation between observed and predicted pCR outcomes. Additionally, the Nagelkerke *R* ^2^ of the model was 0.761, suggesting that 76.1% of the variance in pCR outcomes could be explained by the predictors (ER status, PR status, and HER‐2 status) included in the final multivariate logistic regression model. The model estimation terminated at the seventh iteration due to insufficient change in parameter estimates (< 0.001), confirming the stability of the regression results.

### 3.5. Subgroup Analysis

Given the notably higher pCR rates observed in HER‐2–positive and TNBC subtypes in the overall cohort (Section 3.1), subgroup analyses were further conducted to explore subtype‐specific patterns of NAC response. These analyses focused on stratifying key variables (hormone receptor status, chemotherapy regimen, Ki‐67) within each subtype, with results visualized in Figure 5 (Figure 5a for HER‐2–positive subgroup, Figure 5b for TNBC subgroup).

Figure 5Subgroup analysis of pCR rates in HER‐2‐positive and TNBC Patients. Legend: (a) In HER‐2–positive patients, ER‐negative cases (*n* = 14) had a 93% pCR rate, significantly higher than the 38% rate in ER‐positive cases (*n* = 8) (*χ* ^2^ = 11.75, *p* = 0.001); patients receiving the TCbHP regimen (*n* = 19) had a 79% pCR rate, significantly higher than the 33% rate in the AT regimen group (*n* = 3) (*χ* ^2^ = 9.48, *p* = 0.002). (b) In TNBC patients (*n* = 8), no significant differences in pCR rates were observed between Ki‐67 subgroups (Fisher′s exact test, *p* = 0.625) or chemotherapy regimen subgroups (TP: *n* = 2; TAC: *n* = 3; AT: *n* = 3; Fisher′s exact test, *p* = 0.834). *p* < 0.01 indicates statistical significance.

**Figure:**
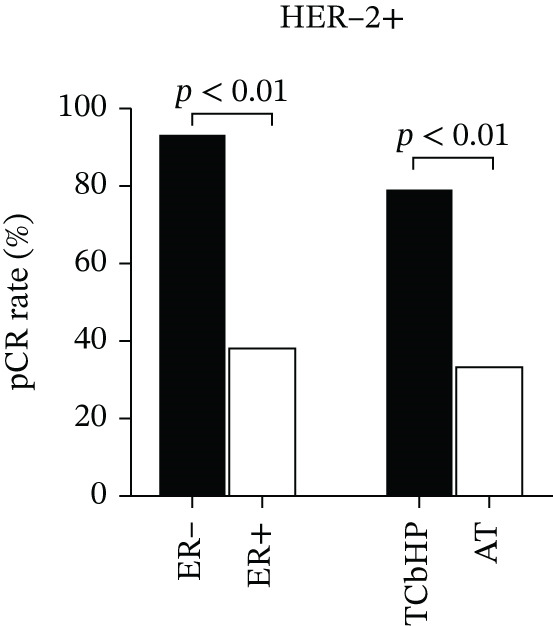
(a)

**Figure:**
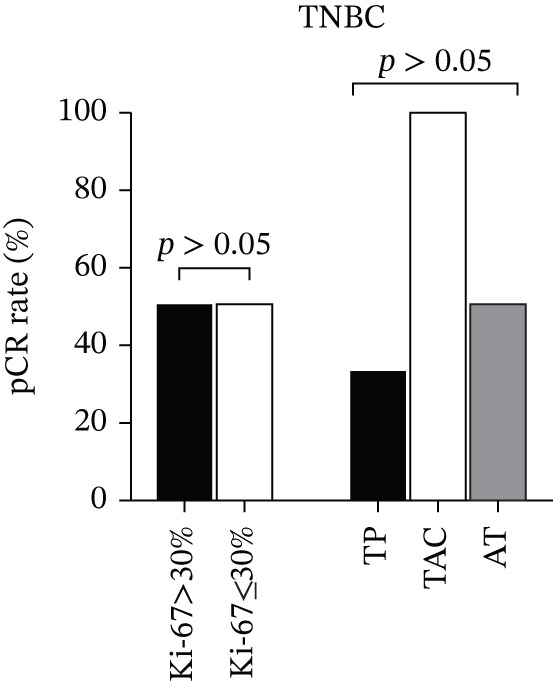
(b)

#### 3.5.1. HER‐2–Positive Subgroup

Specifically, the HER‐2–positive subgroup included 22 patients, accounting for 40.0% of the total cohort. Stratified analysis by ER status revealed a significant difference in pCR rates: ER‐negative patients (*n* = 14) achieved a pCR rate of 93%, whereas ER‐positive patients (*n* = 8) had a markedly lower pCR rate of 38% (*χ* ^2^ = 11.75, *p* = 0.001). This finding supports the negative interaction between ER and HER‐2 signaling identified in the correlation analysis (Section 3.2)—ER negativity appears to eliminate estrogen‐mediated antagonism of the HER‐2/PI3K/AKT pathway, thereby enhancing the therapeutic efficacy of NAC (including dual anti–HER‐2 targeted therapy) in this subtype [12].

Stratification by chemotherapy regimen further confirmed the advantage of dual anti–HER‐2–containing regimens: patients receiving the TCbHP regimen (*n* = 19) had a pCR rate of 79%, which was significantly higher than the 33% pCR rate in those treated with the AT regimen (*n* = 3) (*χ* ^2^ = 9.48, *p* = 0.002). This result supports the clinical value of combining trastuzumab and pertuzumab with chemotherapy for HER‐2–positive breast cancer, as the addition of dual targeted therapy substantially improves the likelihood of achieving pCR compared with chemotherapy alone. Detailed pCR rate distributions across ER status and chemotherapy regimen in this subgroup are presented in Figure 5a.

#### 3.5.2. TNBC Subgroup

The TNBC subgroup included eight patients, accounting for 14.5% of the total cohort. Unlike the significant Ki‐67–pCR association identified in the overall univariate analysis (Section 3.1), stratified analysis by Ki‐67 in this subgroup revealed no meaningful difference in pCR rates: Patients with Ki − 67 > 30*%* (**n** = 5) had a pCR rate of 50%, whereas those with Ki − 67 ≤ 30*%* (**n** = 3) had a pCR rate of 33% (Fisher′s exact test, **p** = 0.625). This inconsistency with the overall cohort is mainly due to the small sample size of the TNBC subgroup—with only five and three patients in the two Ki‐67 strata, the analysis lacked sufficient statistical power to identify subtle differences in pCR rates, even if such differences exist.

Stratification by chemotherapy regimen (TP, TAC, and AT) “further verified no significant associations between chemotherapy regimen and pCR in this subgroup: The TP regimen (**n** = 2) was associated with a 50% pCR rate, whereas the TAC (**n** = 3) and AT (**n** = 3) regimens both had a 33% pCR rate (Fisher′s exact test, **p** = 0.834). Similarly, the limited sample size (≤ 3 patients per regimen) hinders the validation of regimen‐specific efficacy—small group sizes raise the risk of random variation obscuring true therapeutic differences.

Notably, these null findings in this subgroup do not undermine the potential utility of Ki‐67 or specific chemotherapy regimens for TNBC overall; but rather emphasize the need for larger, prospective multicenter cohorts to clarify subtype‐specific response patterns. Detailed distributions of pCR rates across Ki‐67 levels and chemotherapy regimens in the TNBC subgroup are presented in Figure 5b.

## 4. Discussion

pCR has emerged as a critical surrogate endpoint for long‐term survival in breast cancer patients receiving NAC, especially in aggressive subtypes where pCR achievement correlates with improved event‐free and overall survival [18]. This study′s findings align with this clinical paradigm, confirming that post‐NAC pCR rates vary markedly by molecular subtype: HER‐2–positive patients had the highest pCR rate (73.0%), followed by TNBC patients (50.0%), whereas HR‐positive and HER‐2–negative patients showed no pCR cases. Further, multivariate logistic regression identified ER negativity and HER‐2 positivity as independent predictors of higher pCR rates—results that not only support existing evidence on NAC response determinants but also provide subtype‐specific insights to guide individualized clinical decision‐making, particularly for HER‐2–positive and TNBC patients. These observations are consistent with prior work by Spring et al. [10], who reported a pooled pCR rate of 45% in HER‐2–positive and TNBC patients treated with NAC, though our study expands on this finding by clarifying the independent predictive value of ER status alongside HER‐2, a detail not fully addressed in their analysis. Consistent with a recent large‐cohort study by Kang et al. [19], our findings confirm that ER negativity and HER‐2 positivity are independent predictors of pCR in breast cancer patients undergoing NAC—with subtype‐specific pCR rates aligning with their results—thus reinforcing the value of molecular subtype‐guided prediction in clinical practice.

Regarding the nonsignificant CDUS parameters, several potential reasons may account for this. First, the relatively small sample size (*n* = 55) may have restricted the statistical power to detect subtle associations between ultrasound features and pCR—for instance, the CDFI‐related trend (*p* = 0.075) might reach significance in larger cohorts. Second, CDUS parameters such as tumor morphology and aspect ratio are primarily used for preoperative tumor characterization (e.g., benign vs. malignant differentiation) [6], and their utility for predicting chemotherapy response may be inherently limited compared with molecular markers (ER/HER‐2) that directly reflect tumor biological behavior. Third, the high proportion of HER‐2–positive patients (40.0%) in our cohort may have masked the predictive value of ultrasound features, as this subtype is known to have a strong intrinsic response to NAC (especially with targeted therapy) that overrides the contribution of imaging‐based characteristics. Consistent with this, Li et al. [6] reported that CDUS combined with Ki‐67 improved pCR prediction in TNBC, but similar benefits were not observed in HER‐2–positive subtypes—supporting the subtype‐specific utility of imaging parameters.

NAC confers well‐documented benefits for HER‐2–positive and TNBC patients, with pooled pCR rates reaching 45% in prior cohorts [10], and in recent years, the integration of targeted therapy (e.g., trastuzumab + pertuzumab) and immunotherapy has further elevated these rates, underscoring the value of precision strategies for NAC optimization [11, 13, 20–22]; our study further confirmed marked subtype‐specific variation in pCR rates—HER‐2–positive patients showed the highest response, followed by TNBC, consistent with clinical consensus. Subgroup analysis further refined these findings in HER‐2–positive patients: ER‐negative cases had a 93% pCR rate, which was significantly higher than the 38% rate in ER‐positive cases (*χ* ^2^ = 11.75, *p* = 0.001), providing direct support for the “ER/HER‐2 cross talk model” where ER negativity alleviates estrogen‐mediated antagonism of the HER‐2/PI3K/AKT pathway to enhance the synergistic efficacy of dual anti–HER‐2 targeted therapy [23]; additionally, HER‐2–positive patients treated with the TCbHP regimen (containing dual‐targeted therapy) had a 79% pCR rate, a significant improvement over the 33% rate in those receiving the AT regimen (*χ* ^2^ = 9.48, *p* = 0.002), validating targeted therapy as the core component of effective NAC for this subtype. Based on these observations, we propose a precision stratification strategy for HER‐2–positive patients: Prioritize the TCbHP regimen for HER‐2–positive/ER‐negative patients to maximize pCR potential, and consider combining endocrine therapy with dual‐targeted therapy for HER‐2–positive/ER‐positive patients to overcome ER‐mediated drug resistance; in contrast, TNBC patients (*n* = 8) showed no significant associations between Ki‐67 levels or chemotherapy regimens and pCR, a null finding likely attributed to the small sample size (four cases each in the pCR and non‐pCR groups) and limited statistical power, highlighting the need for validation in larger, multicenter cohorts.

Beyond HER‐2–positive and TNBC subtypes, addressing NAC insensitivity in HR‐positive/HER‐2–negative patients is equally critical, as domestic studies report a pCR rate of 3.6% for this subtype, compared with 7%–10% in international cohorts [24, 25]—a discrepancy that may reflect differences in treatment regimens or patient baseline characteristics, and in our cohort, HR‐positive and HER‐2–negative patients accounted for 46% (25/55) with no pCR cases observed, providing indirect evidence for the low responsiveness of this subtype to standard NAC; notably, controversy persists regarding the association between pCR and prognosis in HR‐positive and HER‐2–negative breast cancer [26, 27], but even so, NAC remains clinically valuable for locally advanced cases, as it can achieve a 70% clinical remission rate and 60% breast‐conserving conversion rate—outcomes that facilitate tumor downstaging and expand surgical options [28]. For early‐stage HR‐positive and HER‐2–negative patients (T1–2N0–1M0), data from the SEER database indicate that NAC does not improve survival outcomes [8], and current clinical consensus recommends NAC primarily for patients with tumor size > 5 cm or ≥ 3 lymph node metastases, whereas upfront surgery is preferred for borderline cases (T1–2N0–1M0) [2, 9, 29, 30]; combined with our findings—where HR‐positive and HER‐2–negative patients showed no pCR—we advocate for a comprehensive evaluation of clinical factors (e.g., tumor size, lymph node status, and patient preference) when managing early‐stage HR‐positive and HER‐2–negative patients, with upfront surgery prioritized to maximize clinical benefits and avoid unnecessary NAC‐related toxicity.

This study has several inherent limitations. First, the total sample size was relatively small (*n* = 55) with 20 pCR events. As reported in classic statistical studies [17], EPV < 10 can lead to biased regression coefficients, unstable OR estimates, and extremely wide 95% CIs for ER negativity (1.151–197.543) and HER‐2 positivity (0.000–0.896). Notably, the HER‐2 OR′s 95% CI including 0.000 is biologically inconsistent, contradicting clinical consensus that HER‐2 positivity enhances NAC responsiveness [12, 13, 20], further supporting our findings as hypothesis‐generating.

Second, the single‐center retrospective design with a 20‐month recruitment window (January 2023–August 2024) is prone to selection bias and greatly restricts external validity. On one hand, the molecular subtype distribution in our cohort—with HER‐2–positive patients accounting for 40.0%—is substantially higher than the typical 15%–20% in general breast cancer populations [12, 13], which may be attributed to referral bias: our institution serves as a regional tertiary medical center, attracting HER‐2–positive patients with more aggressive disease from surrounding hospitals for targeted therapy. This subtype enrichment not only potentially introduces spectrum bias but also reflects the unique demographic and clinical characteristics of patients in Nanyang City, Henan Province, rather than the broader breast cancer population. On the other hand, the relatively short recruitment period coincided with the local implementation of the 2023 NCCN Breast Cancer Guidelines, leading to consistent chemotherapy regimen utilization (e.g., preferential use of TCbHP for HER‐2–positive patients) and limiting the diversity of clinical scenarios. Additionally, the 20‐month window restricted the inclusion of patients with rare clinical phenotypes (e.g., triple‐negative breast cancer with BRCA mutations) and reduced the generalizability of our findings to centers with different guideline adherence, treatment preferences, or regional patient demographics. Furthermore, as this is a retrospective study, chemotherapy regimens were not randomly assigned but were determined by clinical factors and physician judgment. Although Table 1 shows no significant correlation between regimen and key baseline variables (e.g., ER/HER‐2 status, N stage), confounding‐by‐indication cannot be completely excluded, which should be considered when interpreting the association between chemotherapy regimen and pCR.

Third, the nonsignificant CDUS–pCR association should be interpreted with caution. The “clinical–imaging–molecular” tridimensional model was proposed to integrate noninvasive imaging features, but the current data do not support the added value of CDUS for pCR prediction. This may be attributed to the small sample size or subtype distribution bias, rather than the inherent lack of utility of imaging parameters. Future studies with larger sample sizes and balanced molecular subtype distribution are needed to reassess the utility of CDUS parameters—such as quantitative ultrasound indices (e.g., vascularity density and elasticity) instead of qualitative classifications—for pCR prediction.

Fourth, LASSO regression was used to address collinearity between clinically correlated variables (e.g., HER‐2 and ER status) and identify core predictors—its application to a small dataset with six variables may be unnecessary, as simpler methods (e.g., stepwise regression) could have yielded comparable results. Future multicenter prospective studies with expanded sample size are needed to increase EPV to ≥ 10 [17], validate the predictive value of ER/HER‐2 status for pCR, mitigate bias, and improve external validity; additionally, these studies should reevaluate the utility of CDUS parameters—such as quantitative ultrasound indices (e.g., vascularity density, elasticity) instead of qualitative classifications—for pCR prediction, and focus on borderline luminal‐type breast cancer patients (e.g., T1–2N0–1M0), a subgroup with controversial NAC utility.

## 5. Conclusion

pCR rates show significant variability across breast cancer molecular subtypes following NAC, with ER negativity and HER‐2 positivity emerging as independent predictors of higher pCR rates. These findings highlight the need to integrate baseline characteristics—including ER/HER‐2 status, tumor stage, and lymph node involvement—into clinical decision‐making to identify optimal NAC candidates. By tailoring treatment strategies to subtype‐specific biological features, clinicians can enhance therapeutic efficacy and optimize outcomes for breast cancer patients, particularly in HER‐2–positive/ER‐negative and triple‐negative subtypes where NAC responsiveness is most pronounced.

## Author Contributions

Yadong Zhang (first author): conceptualization, data collection, and development of methodology; Shubian Qiu: data curation and literature review; Xin Wang (corresponding author): project administration and writing of the original manuscript draft.

## Funding

This study was supported by the Science and Technology Research Project of Nanyang City (24KJGG242).

## Disclosure

Xin Wang critically revised the manuscript for important intellectual content.

## Ethics Statement

This study was reviewed and approved by the Ethics Committee of Nanyang Second People′s Hospital (Approval No. 2024‐LY025‐01‐K01), in accordance with the Declaration of Helsinki.

## Consent

All patients or their legal representatives provided written informed consent prior to study enrollment for the use of their clinical data in this study.

## Conflicts of Interest

The authors declare no conflicts of interest.

## Data Availability

The datasets used and/or analyzed in the current study are available from the corresponding author upon reasonable request for academic purposes.
